# Production and Sensing of Butyrate in a Probiotic *E. coli* Strain

**DOI:** 10.3390/ijms21103615

**Published:** 2020-05-20

**Authors:** Yanfen Bai, Thomas J. Mansell

**Affiliations:** Department of Chemical and Biological Engineering, Iowa State University, Ames, IA 50011, USA; baifen118@gmail.com

**Keywords:** metabolic engineering, *E. coli* Nissle 1917, short-chain fatty acids, biosensor

## Abstract

The short-chain fatty acid butyrate plays critical roles in human gut health, affecting immunomodulation, cell differentiation, and apoptosis, while also serving as the preferred carbon source for colon cells. In this work, we have engineered a model probiotic organism, *E. coli* Nissle 1917 (EcN, serotype O6:K5:H1), to produce butyrate from genomic loci up to approximately 1 g/L (11 mM). Then, for real-time monitoring of butyrate production in cultures, we developed a high-throughput biosensor that responds to intracellular butyrate concentrations, with green fluorescent protein as the reporter. This work provides a foundation for studies of butyrate for therapeutic applications.

## 1. Introduction

Short-chain fatty acids (SCFAs) such as butyrate have an important role in the mammalian gut. In this environment, butyrate can affect immunomodulation [1], cell differentiation [2], apoptosis, and autophagy [3,4]. In addition, butyrate is the preferred carbon source for colon cells [5]. A recent study proposed that butyrate-producing clostridia produce butyrate for use by colonocytes, because its rapid consumption depletes oxygen, maintaining hypoxic conditions, and thereby limiting the growth of aerobic pathogens such as *Salmonella* [6].

Butyrate is normally produced in the gut by butyrogenic anaerobic bacteria (e.g., *Faecalibacterium prausnitzii* [7] or *Clostridium spp*. [8]). However, low-fiber diets can decrease the cross-feeding of these species [9], resulting in low levels of gut butyrate, which may lead to inflammation and other bowel diseases. Butyrogenic bacteria populations are also decreased in many inflammatory bowel diseases [10]. Given the importance of butyrate to gut health, pilot studies of the administration of butyrate have been performed via oral [11,12,13] and rectal routes [14]. These therapies have several limitations, including the release of butyrate from enteric capsules outside the intended location [15], as well as the disagreeable odor and taste of butyrate (“vomit” is a common descriptor for butyrate and its esters). As such, the study of the effect of butyrate in the gut environment is difficult to undertake, as reliable delivery of butyrate is challenging [16].

Probiotics are live microorganisms that provide a benefit to the host [17]. *E. coli* Nissle 1917 (EcN, serotype O6:K5:H1) is one well-known probiotic candidate that has been used for decades as an over-the-counter remedy for traveler’s diarrhea in Canada and Europe [18,19]. While most probiotic research has focused on the administration of microbes found in nature, some probiotics have been engineered to execute specific therapeutic activities, including delivery of antitumor drugs and insulin for cancer therapy and diabetes [20,21,22], secretion of antimicrobial peptides [23] and signaling molecules [24], production of enzymes [25], and accelerating wound healing [26]. Most recently, a strain of EcN engineered to metabolize phenylalanine in the gut of phenylketonurics [27] has entered phase 1/2a clinical trials [28].

Previously, *E. coli* (*E. coli*) has been genetically engineered to produce SCFAs [29,30,31]. However, most of these studies focused on increasing the production of SCFAs for industrial applications and relied on the use of plasmids, which usually require the use of antibiotics for maintenance. To this end, we constructed a butyric-acid-producing *E. coli* Nissle 1917 (EcN) strain by CRISPR-Cas9-assisted knockout of electron acceptor pathways and heterologous genomic expression of butyric acid (C4) production enzymes. Furthermore, to facilitate real-time monitoring of C4 levels in situ, a sensitive C4-responsive biosensor was developed in this study. 

## 2. Results

### 2.1. Construction of a Butyrate Biosynthetic Pathway in E. coli Nissle 1917

**Figure 1:**
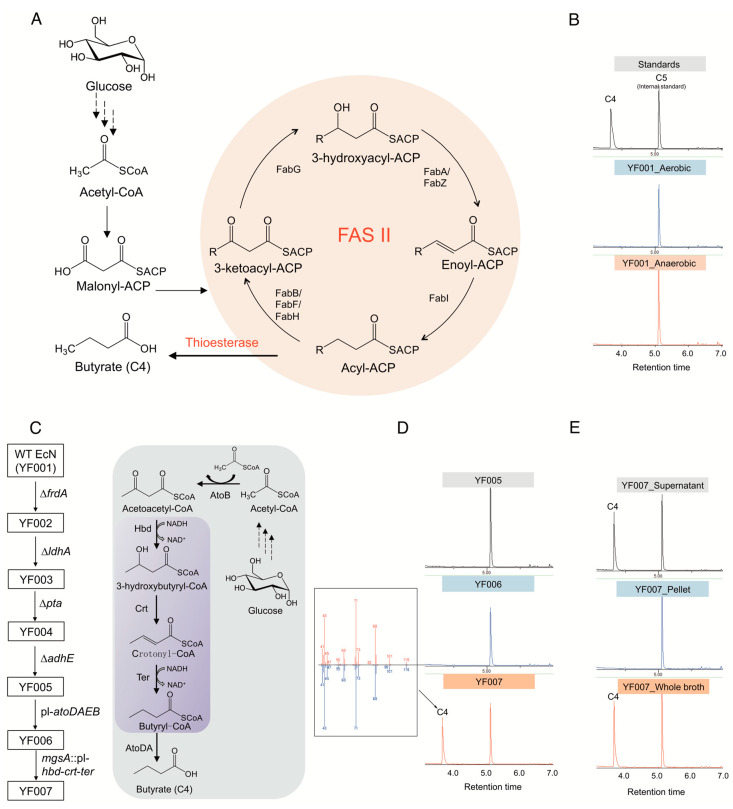
Synthesis of butyrate in *E. coli* Nissle 1917 (EcN). (**A**) Butyrate production mediated by using *E. coli* native type II fatty acid biosynthetic pathway (FAS II). (**B**) Gas chromatography (GC) profile of fermentation broth of wild type (WT) EcN strain (YF001) cultured under both aerobic and anaerobic conditions. (**C**) Construction of C4 chassis strain by disruption of byproduct-competitive pathways and introduction of semiheterologous pathway. Purple box indicates the heterologous genes expressed. (**D**) Gas chromatography (GC) profile of the engineered EcN strains YF005, YF006, and YF007. The C4 peak was further verified by mass spectrometry (MS). (**E**) C4 distribution in the engineered strain YF007.

The type II fatty acid biosynthetic (FAS II) pathway has been recognized as the primary route for fatty acid production in *E. coli* (Figure 1A). However, we found that wild-type EcN does not natively produce detectable butyrate under either aerobic or anaerobic conditions (Figure 1B). 

Here, the competitive pathways were disrupted before construction of the C4 biosynthesis pathway (Figure 1C). To this end, genes involved in biosynthesis of these byproducts, i.e., *frd*A, *ldh*A, *adh*E, and *pta*, were deleted using iterative CRISPR-Cas9-assisted recombination [32] in wild-type EcN (or YF001), resulting in the quadruple knockout chassis strain EcN YF005 (∆*frd*A, ∆*ldh*A, ∆*adh*E, ∆*pta*) (Figure 1C). Analysis by gas chromatography-mass spectrometry (GC-MS) of cell cultures showed that YF005 was still not able to synthesize C4 (Figure 1D), which demonstrates that EcN does not natively produce C4 through the FAS II pathway without further engineering efforts [33].

Next, construction of a route to exclusively produce C4 was considered. In contrast to *E. coli*, many microbes such as *Clostridium tyrobutyricum*, *C. beijerinckii*, and *C. thermobutyricum* are known to be able to synthesize C4, and the biosynthetic pathway has been elucidated in prior studies [34,35]. Briefly, two molecules of acetyl-CoA are condensed by thiolase AtoB to yield acetoacetyl-CoA, which can be further catalyzed by the 3-hydroxybutyryl-CoA dehydrogenase Hbd to form 3-hydroxybutyryl-CoA. After dehydration catalyzed by 3-hydroxybutyryl-CoA dehydratase Crt and reduction reaction catalyzed by trans-enoyl-CoA reductase Ter, butyryl-CoA is formed. Finally, acetate CoA-transferase AtoDA catalyzes the removal of the CoA group from butyryl-CoA, resulting in the formation of butyrate (Figure 1C).

In this study, we conceived a semiheterologous pathway and applied it to EcN. Specifically, *E. coli* endogenous AtoB (the first pathway enzyme) and AtoDA (the last pathway enzyme) were overexpressed by replacing the native promoter of the *atoDAEB* operon with a strong, constitutive P_L_ promoter from phage λ [36] (Figure 1C). In addition, *hbd* and *crt* from *Clostridium acetobutylicum* [37] and *ter* from *Treponema denticola* [38] were integrated in the genome of EcN, replacing the *mgs*A gene, a known safe site for integration [39], with expression controlled by another P_L_ promoter. After the above manipulations, the engineered EcN YF007 strain (YF005, PL-*ato*DABE, PL-*hbd*-*crt*-*ter*) (Figure 1C) was cultured by microaerobic fermentation for 48 h in Luria-Bertani (LB) media supplemented with 2% (*w*/*v*) glucose. After fermentation, the culture was spiked with valeric acid (C5) and derivatized to create ethyl esters of fatty acid analysts. We noted a peak from the fermentation broth of YF007 with the same retention time as ethyl butyrate standard (T*_R_* = 3.62 min) (Figure 1D). Further mass spectrometry data confirmed the identity of C4 produced from YF007 (Figure 1D), which demonstrated the successful biosynthesis of C4 from the constructed pathway. Moreover, we found that almost all of the C4 was present in the supernatant rather than the cell pellet (Figure 1E), indicating that the produced butyrate was secreted.

### 2.2. Effects of Inducible Expression System and Different Carbon Sources on C4 Production

**Figure 2:**
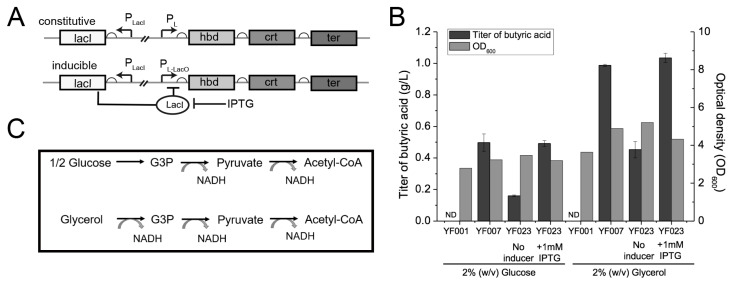
Optimization of conditions for C4 production. (**A**) Constitutive and inducible expression systems for C4 production. (LacI, lac repressor; LacO, lac operator.) (**B**) C4 titers and cell mass (optical density at 600 nm [OD_600_]) of the engineered strains YF007 and YF023 under different conditions. (ND, not detected). (**C**) Comparison of catabolism of glycerol and glucose for supply of NADH reducing equivalent.

Overproduction of SCFAs can cause toxicity to the host strain [40,41], leading to membrane damage, and thus compromising the host strain’s performance. Therefore, we used an inducible expression system to express the C4 synthesis pathway. Specifically, a lacO operator was inserted between the P_L_ promoter and the open reading frames (ORFs) of the *hbd*-*crt*-*ter* gene cluster in YF007 to obtain isopropyl β-D-1-thiogalactopyranoside (IPTG)-dependent P_L-lacO_ for inducible expression in strain YF023 (P_L-lacO_-*hbd*-*crt*-*ter*) (Figure 2A). Next, both systems were characterized for C4 production. When characterized in LB containing 2% (*w*/*v*) glucose, no significant increases in cell density or C4 titer were observed (Figure 2B). Specifically, YF023 produced 0.49 ± 0.02 g/L C4, which is comparable to YF007 (0.50 ± 0.06 g/L). 

As the described pathway for C4 biosynthesis is NADH-dependent, i.e., for synthesis of one molecule of C4, two molecules of NADH are consumed [42] (Figure 2C), we reasoned that increasing the supply of NADH might improve C4 yield and final titers. Compared to glucose, glycerol has a higher degree of reduction; glycerol generates a reducing equivalent with yields of 3 mol NADH per mol, while glucose generates only 2 mol NADH under the same carbon equivalent (Figure 2C). Thus, here glycerol was employed as the carbon source for C4 production. In agreement with our hypothesis, when cultured in LB supplemented with 2% (*w*/*v*) glycerol, YF007 produced 0.99 ± 0.01 g/L C4, which is approximately 100% higher than using glucose as the carbon source (LB + 2% glucose, 0.50 ± 0.06 g/L). Consistently, YF023 also produced 1.03 ± 0.03 g/L C4, which increased by ~110% in contrast to using glucose (0.49 ± 0.02 g/L) (Figure 2B), which confirms our hypothesis that increasing the supply of the reducing equivalent contributes to C4 production.

### 2.3. Construction of High-Throughput Butyrate Sensor System in EcN

**Figure 3:**
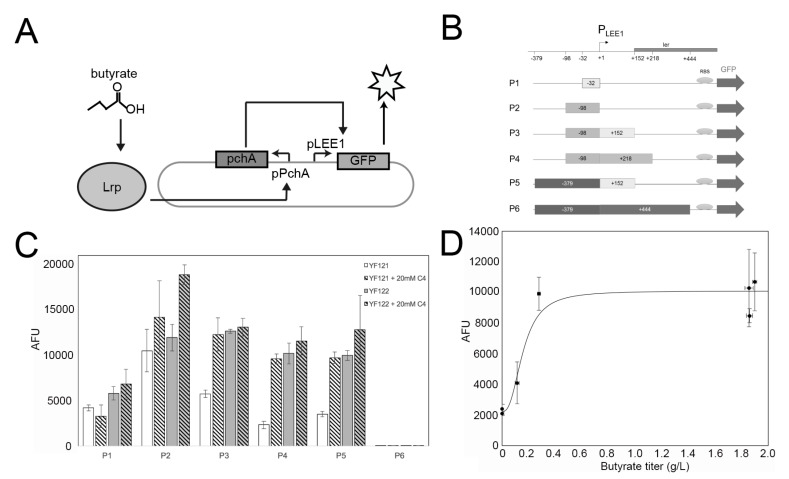
Construction of C4 sensor system in EcN. (**A**) Schematic of the in vivo butyrate sensor system in EcN. (**B**) Six different reporter plasmids (P1-P6) with distinct lengths of P_LEE1_ promoters; +1 is the transcription start site (TSS). (**C**) Fluorescent signal intensities of the YF121 (negative control) and YF122 (test) strains with plasmids P1-P6, cultured in the absence or presence of 20 mM sodium butyrate (C4). The data were calculated from at least three biological replicates. (**D**) Fluorescence measurements with respect to butyrate titer for seven different growth conditions; strains YF121, YF122, and YF126 containing plasmid P4 were cultured with and without 20 mM exogenous sodium butyrate. In addition, YF126 was cultured with isopropyl-β-D-thiogalactoside (IPTG) and no added butyrate).

Nakanishi et al. discovered that toxin secretion in the enterohemorrhagic *E. coli* strain O157:H7 (Sakai) is responsive to butyrate concentration [43]. Briefly, they found that the native *E. coli* leucine-responsive regulatory protein (Lrp) forms a binary complex with C4, which can bind to the promoter region of *pch*A (P_pchA_) and then initiate the transcriptional expression of *pch*A. The PchA regulator is able to bind to the promoter of the locus of enterocyte effacement (P_LEE1_), activating transcription of the *ler* gene under control of P_LEE1_ [44] (Figure 3A). Inspired by this finding, here we employed these interactions and attempted to develop the C4 biosensor by replacing the *E. coli ler* gene by the *gfp* gene, which encodes a green fluorescent protein (GFP) reporter under the P_LEE1_ promoter. Herein, we amplified the necessary parts from O157:H7 genomic DNA to create recombinant plasmid pDMB-PpchA-*pch*A-P_LEE1_-*gfp* (Figure 3B). Additionally, it should be noted that the precise PchA binding site to the P_LEE1_ promoter is still unclear [44]. Therefore, we synthesized intergenic regions of different lengths as P_LEE1_ candidates (−32 bp, −98 bp, −98 + 152 bp, −98 + 218 bp, −379 + 152 bp, −379 + 444 bp), creating 6 plasmids (pC4S-P1 to pC4S-P6, Figure 3B). The engineered non-butyrate-producing strain YF006 (YF 005 P_L_-*ato*DABE) and butyrate-producing strain YF007 (YF 005 P_L_-*ato*DABE, P_L_-*hbd*-*crt*-*ter*) were initially used as the host strains for sensor system testing. However, initial results showed that although the GFP signal was successfully detected in expression strain YF007, it is not obviously distinct from YF006.

We proposed that the expression of genes in this region might be susceptible to native *E. coli* endogenous regulation. For instance, it has been reported that H-NS, encoded by the *hns* gene, which is an *E. coli* global transcriptional silencer, can regulate ~5% of all *E. coli* gene expression [45,46]. Subsequently, the *hns* gene was deleted in YF007, resulting in YF122 (YF007 ∆*hns*), while its deletion in YF006 resulted in control strain YF121 (YF006 ∆*hns*). We again applied the six sensor plasmids to the two ∆*hns* strains. Consistent with our hypothesis, ∆*hns* deletion led to butyrate-dependent fluorescent expression in YF121 and YF122 (Figure 3C). For most plasmids, YF122 exhibited significant differences to YF121. Among such plasmids, pC4S-P4 (pDMB-PpchA-*pch*A-P_LEE1_ (−98 + 218 bp)-*gfp*) enabled YF122 to exhibit more than a five-fold difference compared with YF121 (*p* < 0.01) (Figure 3C). Next, we further examined the relationship between C4 titers and GFP signal intensities in YF122-containing pC4S-P4. The results indicated that the sensor exhibited a K_1/2_ (the input concentration where the transcriptional output is half-maximal) detection threshold of about 0.1 g/L and had approximately 5-fold change in signal from 0 to 0.28 g/L, which represents relatively robust C4 detection using biosensors.

## 3. Discussion

The wild-type EcN does not natively produce detectable butyrate, which is not surprising because the *E. coli* native FAS II pathway usually produces long-chain fatty acids (LCFAs, e.g., C16:0, C18:1) for biosynthesis of membrane phospholipids [47]. Prior studies have shown that C4 biosynthesis can be achieved by *E. coli* through expression of a heterologous thioesterase (e.g., TesAT from *Anaerococcus tetradius*), as well as through using the cerulenin inhibitor, which restricts the fatty acid elongation cycle. Nevertheless, the promiscuity of the thioesterase led to production of other SCFAs, such as C6, C8 and C10 [33]. It should be also noted that under anaerobic conditions, wild-type *E. coli* is capable of mixed acid fermentation, i.e., formic acid, succinate, lactate, ethanol. and acetate are also synthesized from carbon sources (e.g., glucose) [48]. Synthesis of these byproducts creates competition for common precursors (e.g., pyruvate, acetyl-CoA) and reducing cofactors (e.g., NAD(P)H) [48].

Here, we have presented a semiheterologous pathway for C4 production in EcN. All parts of the pathway were inserted into the genome, eliminating the need for plasmids for butyrate production. We created this strain using CRISPR-Cas9-assisted knockout and insertion technologies. As of this writing, we believe this is the first use of CRISPR-Cas9 to modify the genome of EcN, demonstrating that this technology can be used to rapidly engineer this strain. Butyrate was specifically produced, while other SCFAs such as C6 and C8 were not detectable, making the effect of C4 clear for future therapeutic applications. We also noticed that most of the C4 was found to be present in the supernatant instead of the cell pellet, which is consistent with prior studies showing that SCFAs produced by *E. coli* were primarily found in the extracellular milieu [49]. These findings suggest that the presence of a SCFA high-efficiency transport mechanism across the cellular membrane facilitates the export of C4, while effluxes such as the AcrAB-TolC complex might be responsible for the export of intracellular fatty acids produced by *E. coli* [49]. Butyrate may cause toxicity to the host strain when produced. In this study, we attempted to alleviate this toxicity by developing an inducible expression system. However, no further increases in cell growth or C4 titers were observed. There are two possibilities for this: First, C4 is not as toxic as other SCFAs, such as C6, C8 and C10. Prior have studies have also shown that there is an association between SCFA toxicity and SCFA chain length, whereby an increase in length enables higher toxicity [40]. For an organic solvent, toxicity is often associated with hydrophobicity. Second, we utilized rich media for C4 production in this study. The nutrients provided by the LB-rich medium might compensate for the damage caused by the fatty acids compared with the minimal medium [41].

Furthermore, in this study, glycerol was found to be a more efficient substrate than glucose for C4 production in the LB medium as more NADH is produced from glycerol catabolism. Alternatively, expression of C4 biosynthetic pathways might be repressed by glucose via catabolite repression [50], which could explain the lower C4 production with added glucose in IPTG-inducible strains.

For some chemicals or drugs, a low dose has a stimulative or beneficial effect, while a high dose causes inhibitory or toxic effects, a phenomenon known as hormesis [51]. Excessive secretion of C4 might also compromise its therapeutic effect; therefore, construction of a sensor system to monitor and fine-tune the levels of C4 is highly desirable. We attempted to create a genetic circuit to link the fluorescent protein signal with the level of intracellularly produced butyrate.

Our C4 sensor system exhibited high sensitivity to C4 dosage (as low as 0.1 g/L) and has the potential to be applied for C4 detection in the future. We also noticed that the dynamic range of this sensor was relatively narrow and the sensor platform reached saturation after 0.28 g/L. In the future, two strategies could be employed to further engineer the sensor. First, the high basal output could be lowered, which can be achieved by tighter binding between the regulator and promoter [52]. Second, the maximal output could be increased, which can be done by tuning the regulator expression level or adjusting the promoter strength [52]. Additionally, the production of C4 could be controlled by creating a feedback loop in which the transcription *hbd*-*crt*-*ter* gene cluster is decreased by a repressor under control of the P_LEE1_ butyrate-responsive promoter.

We also noticed that among the six GFP plasmids, pC4S-P6, which harbors the P_LEE1_ promoter candidate from −379 to +444 bp, abolished the GFP signal intensity completely, which might be due to transcriptional repression. This is because P5 (harboring the P_LEE1_ promoter from −379 + 152 bp) exhibited comparable signal intensity to P4 (P_LEE1_ promoter from −98 + 218 bp), which suggests the presence of the fragment ranging from −379 to −98 does not greatly affect the transcriptional expression of *gfp*. Instead, the promoter region ranging from +218 to +444 bp may impose a detrimental effect on the transcription of *gfp*, which may be involved in the formation of RNA secondary structure that causes RNA polymerase to pause. Additionally, the best performing plasmid, pC4S-P4, contains a small part of the *ler* gene in the P_LEE1_ promoter region. It is not clear what this fragment’s role is in transcriptional regulation of the operon.

In summary, here we constructed a C4-producing engineered probiotic. In addition, a high-sensitivity C4 sensor system was constructed to monitor the in vivo butyrate level. We believe both will have wide applications in the future—the C4-producing engineered probiotic can be used as a butyrate source for gut health, while the sensor system can be used for both high-throughput screening for butyrate-producing strains, and can be used for real-time monitoring of butyrate titers in vivo.

## 4. Materials and Methods

### 4.1. Bacterial Strains, Plasmids, and Culture Conditions

All plasmids and *E. coli* strains used in this study are listed in Table 1. *E. coli* DH5α was used as a cloning host. EcN was kindly provided by Dr. Milton Allison (Iowa State University College of Veterinary Medicine). The plasmids pCas and pTargetF were kindly supplied by Dr. Sheng Yang (Addgene plasmids 62225 and 62226). *E. coli* strains were cultured in Luria–Bertani (LB) medium at 37 °C or 30 °C, except for biosensing experiments, in which EcN strains were cultured in Dulbecco’s modified Eagle’s medium (DMEM) (Thermo Fisher Scientific, Waltham, MA, USA). Kanamycin (50 mg/L), spectinomycin (50 mg/L), ampicillin (100 mg/L), chloramphenicol (25 mg/L), and Isopropyl-β-d-thiogalactoside (IPTG) (0, 0.01, 0.1, 0.5, 1 mM) were supplemented to the medium when needed. Product standards, including butyric acid (C4), valeric acid (C5), and sodium butyrate, were purchased from Sigma-Aldrich (Saint Louis, MO, USA). 

### 4.2. Strain Construction

Iterative CRISPR-Cas genome editing technology [32,53] was employed to construct the EcN strain with *frd*A/*ldh*A/*adh*E/*pta* knockouts. The use of CRISPR-Cas9 required (1) guide RNA constructs corresponding to each knockout or insertion point and (2) dsDNA donors for recombineering. Sequences encoding various guide RNAs were inserted into plasmid pTargetF by DNA assembly kit and sequences of the oligos used are available in Table S1. Donor DNA for knockouts constituted a 1000 bp substrate representing the regions 500 bp upstream and downstream of the gene to be knocked out. For recursive CRISPR-assisted knockouts or insertions, EcN-harboring pCas was first cultured in LB with kanamycin at 30 °C for 2 h, supplied with 2% (*w/v*) arabinose to induce the λ-red recombination system. Then, cell pellets were collected, washed, and suspended in 10% (*v*/*v*) cold sterilized glycerol. Next, 100 ng of pTargetF plasmid expressing the gRNA of the target gene and 400 ng donor DNA were transfected into EcN electroporation cells. After electroporation, 1 mL SOC medium was supplied for recovery growth at 30 °C for 1 h. Finally, the recovery culture was spread onto the LB agar plate with spectinomycin and kanamycin and incubated at 30 °C overnight. The positive transformants were isolated and verified by colony PCR. Next, the pTargetF plasmid was cured by 1 mM IPTG at 30 °C to obtain a pTargetF-free strain, which served as the host strain for the next round of gene deletion. The order of knockouts was Δ*frd*A, Δ*ldh*A, Δ*adh*E, and Δ*pta* to obtain the EcN quadruple knockout designated YF005 (Table 1).

Next, the native promoter of the atoDAEB operon in the YF005 strain was replaced with the strong constitutive PL promoter from bacteriophage λ [36], resulting in strain YF006. Donor DNA in this case comprised the PL promoter flanked by 500 bp fragments up- and downstream of the *E. coli* atoDAEB promoter. To insert the genes for butyrate production, the *hbd* and *crt* genes from *Clostridium acetobutylicum* (ATCC #824D-5) and the *ter* gene from *Treponema denticola* (ATCC 35405D-5) were amplified from relevant genomic DNA with primers hbd-up/hbd-down, crt-up/crt-down, and ter-up/ter-down. We synthesized the PL promoter in the primer hbd-up; the sequences of PL are shown in the Table S1 in the Supplemental Material. These three genes were combined by Gibson assembly [56] as an operon with the native ribosome binding site (RBS) of each gene, and the constitutive P_L_ or inducible P_L-LacO_ was added before the *hbd* gene, as shown in Figure 1.

Finally, the P_L_-*hbd-crt-ter* and P_L-LacO_-*hbd-crt-ter* operons were individually inserted into the genome of strain YF006 at the mgsA safe site, resulting in strains YF007 and YF023, respectively (Table 1). The *pyrF* gene in strains YF001 and YF007 was deleted as above to obtain strains YF021 and YF022, respectively. For the construction of the butyrate sensor system, the *hns* gene in strains YF006, YF007, and YF023 was also removed as above, resulting in recombinant strains YF121, YF122, and YF126, respectively (Table 1).

### 4.3. Biosensor Plasmid Construction

For construction of pDMB-PpchA-pchA-PLEE1-GFP series plasmids, the open reading frame (ORF) of *pch*A and its native promoter PpchA from enterohemorrhagic *E. coli* were de novo synthesized as a gBlock (Integrated DNA Technologies, Coralville, IA, USA) and cloned between the SspI and HindIII restriction sites of vector pDMB using Gibson assembly. The pDMB plasmid is a chloramphenicol-resistant derivative of pTrc99A (Amersham Biosciences, Corston, UK) [55]. PLEE1 intergenic regions of various lengths with different lengths were PCR-amplified from genomic DNA of enterohemorrhagic *E. coli* Sakai (O157:H7) purchased from the American Type Culture Collection (Manassas, VA, USA) (BAA-460D-5). GFP was amplified from pET28a-GFP, which was a gift from Matthew Bennett (Addgene plasmid # 60733) [54].

### 4.4. Butyrate Production by E. coli Nissle 1917

Individual colonies were picked up from Luria–Bertani Broth (LB) plates and then inoculated into 3 mL of LB liquid medium at 37 °C and 250 rpm overnight for seed culture preparation. Then, the seed culture was added to 3 mL LB with 2% (*w*/*v*) glucose or LB with 2% (*w*/*v*) glycerol at a final OD_600_ of approximately 0.1. For the inducible system, isopropyl-β-D-thiogalactopyranoside (IPTG) inducer was typically added at a final concentration of 1 mM during the mid-log phase (OD_600_ 0.6–0.8). For optimization of IPTG dosages, final concentrations of 0, 0.01, 0.1, 0.5, and 1 mM IPTG were used.

### 4.5. Butyrate Extraction and Detection by GC-MS

One milliliter of either the whole liquid media sample, cell pellet, or supernatant was harvested and 125 µL 10% (*w/v*) NaCl and 125 µL acetic acid were added into the sample. Then, 20 µL internal standard (25 g/L valeric acid in ethanol) was added into the sample, followed by 500 µL ethyl acetate. The mixture was then vortexed for 30 s and centrifuged at 14,000 rpm for 10 min. Next, 250 µL of the top organic layer was transferred into a fresh glass tube and 2.25 mL ethanol/hydrochloric acid (30:1, v/v) was added, vortexed again, and then incubated at 55 °C for 1 h. Upon completion, an additional 1.25 mL ddH2O and 1.25 mL hexane were added and the mixture was vortexed for 30 s, then centrifuged at 2000 g for 2 min. Finally, the top hexane layer was collected and analyzed by GC-MS. The temperature for GC-MS analysis was initially held at 50 °C for 2 min, ramped to 200 °C at 25 °C/min, held for 1 min, then raised to 315 °C at 25 °C/min and held for 2 min. The carrier gas was helium and the flow rate was set at 1 mL/min through a DB-5MS separation column (30 m, 0.25 mm ID, 0.25 μm, Agilent, Santa Clara, CA, USA).

### 4.6. Detection of Sensor System of Butyrate

The butyrate-producing EcN strains YF122 and YF126 with the plasmid pC4S-P4 were cultured overnight in DMEM medium (Thermo Fisher Scientific, Waltham, MA, USA), with and without 20 mM sodium butyrate at 37 °C and 250 rpm. The cultures were collected and diluted to an OD_600_ of approximately 0.1 with PBS buffer (pH 7.0), then analyzed by a BD Biosciences FACSCanto II flow cytometer for detection of GFP, as previously described [57]. Experiments were repeated at least three times and the standard errors were calculated. The non-butyrate-producing strain YF121 harboring pC4S-P4 was employed as negative control.

## Figures and Tables

**Table 1:** Strains and plasmids used in this study with antibiotic resistance (e.g., Kan^R^) (Kan: kanamycin, Spe: spectinomycin, Cm: chloramphenicol).

Plasmids/Strains	Genetic Characteristics	Source
**Plasmids**		
pCas	repA101(Ts), P_cas_-cas9, P_araR_-Red lacI^q^, P_trc_-sgRNA-pMB1; Kan^R^	[53]
pTargetF	pMB1, *aad*A; Spe^R^	[53]
pTargetF-*mgs*A	pMB, sgRNA-*mgs*A; Spe^R^	This study
pTargetF-*frd*A	pMB1, sgRNA-*frd*A; Spe^R^	This study
pTargetF-*ldh*A	pMB1, sgRNA-*ldh*A; Spe^R^	This study
pTargetF-*pta*	pMB1, sgRNA-*pta*; Spe^R^	This study
pTargetF-*adh*E	pMB1, sgRNA-*adh*E; Spe^R^	This study
pTargetF-*ato*DA	pMB1, sgRNA-*ato*DA; Spe^R^	This study
pTargetF-*hns*	pMB1, sgRNA-*hns*; Spe^R^	This study
pET28a-GFP	pBR322 ori with P_T7_; GFP; Kan^R^	[54]
pDMB	Modified pTrc99A containing ssDsbA and *bla*; Cm^R^	[55]
pC4S-P1	pDMB,PpchA-*pch*A-P_LEE1(−32 bp)_-*gfp*; Cm^R^	This study
pC4S-P2	pDMB,PpchA-*pch*A-P_LEE1(−98 bp)_-*gfp*; Cm^R^	This study
pC4S-P3	pDMB,PpchA-*pch*A-P_LEE1(−98 + 152 bp)_-*gfp*; Cm^R^	This study
pC4S-P4	pDMB,PpchA-*pch*A-P_LEE1(−98 + 218 bp)_-*gfp*; Cm^R^	This study
pC4S-P5	pDMB,PpchA-*pch*A-P_LEE1(−379 + 152 bp)_-*gfp*; Cm^R^	This study
pC4S-P6	pDMB,PpchA-*pch*A-P_LEE1(−379 + 444 bp)_-*gfp*; Cm^R^	This study
***E.coli* Strains**		
YF001	*E.coli* Nissle 1917, wild-type	Gift of Milton Allison, Iowa State University
YF005	*E.coli* Nissle 1917, ∆*frd*A, ∆*ldh*A, ∆*adh*E, ∆*pta*	This study
YF006	YF005, P_L_-*ato*DABE	This study
YF007	YF006, *mgs*A::P_L_-*hbd*-*crt*-*ter*	This study
YF023	YF006, *mgs*A::P_L-LacO_-*hbd*-*crt*-*ter*	This study
YF021	YF001, ∆*pyr*F	This study
YF022	YF007, ∆*pyr*F	This study
YF121	YF006, ∆*hns*	This study
YF122	YF007, ∆*hns*	This study
YF126	YF023, ∆*hns*	This study

## Supplementary Materials

Supplementary materials can be found at https://www.mdpi.com/1422-0067/21/10/3615/s1.
