# Supplementary material for: Production and Sensing of Butyrate in a Probiotic E. coli Strain
Source: Int J Mol Sci. 2020 May 20;21(10):3615. doi: 10.3390/ijms21103615 (PMC7279287; doi:10.3390/ijms21103615)
Supplement: Supplementary file 1 [file ijms-21-03615-s001.pdf]

Supplementary materials:

Table S1. Primers used in this study.

Name	Squences (5'-3')
**gRNA-up**	ACTAGTATTATACCTAGGACTGAG
**gRNA-down-mgsA**	GTCCTAGGTATAATACTAGTAACGTCAACGCGATGTTGAGGTTTTAGAGCTAGAAATAGC
**gRNA-down-frdA**	GTCCTAGGTATAATACTAGTAAATGACCCAACTGGAACTGGTTTTAGAGCTAGAAATAGC
**gRNA-down-ldhA**	GTCCTAGGTATAATACTAGTAAAACCGCTAAAACTGCCAAGTTTTAGAGCTAGAAATAGC
**gRNA-down-pta**	GTCCTAGGTATAATACTAGTACGATAGTCGTAGTCTGATCGTTTTAGAGCTAGAAATAGC
**gRNA-down-adhE**	GTCCTAGGTATAATACTAGTGACTCTGGGTTGTGGTTCCTGTTTTAGAGCTAGAAATAGC
**gRNA-pl-promoter-atoD-down**	GTCCTAGGTATAATACTAGTAACATTCAATAATCTCATTCGTTTTAGAGCTAGAAATAGC
**gRNA-H-NS-down**	GTCCTAGGTATAATACTAGTTTACAGCTGGAGTACGGCCCGTTTTAGAGCTAGAAATAGC
**frdA-L-up**	ACTGGTGATACTTCGCCATC
**frdA-L-down**	CACTAATCTCAAAAGTATACCGGTGGCGAAGCGGATGC 阿 AG
**frdA-R-up**	CTGCATCCGCTTCGCCACCGGTATACTTTTGAGATTAGTG
**frdA-R-down**	AGTTAGGAATGGATGCGCTC
**ldhA-L-up**	ATCATACGGGTCATTGCCAG
**ldhA-L-down**	GTCCTTTGGCTTTGAGCTGGATTCCTGACAGCAGAAGCTC
**ldhA-R-up**	GAGCTTCTGCTGTCAGGAATCCAGCTCAAAGCCAAAGGAC
**ldhA-R-down**	CCCACCAGATAACGGAGATC
**adhE-L-up**	GTCTGAATAACGGCACTG
**adhE-L-down**	CACTCAAGAGCAAGTAGACACTACGGTCGTGATTATGTAG
**adhE-R-up**	CTACATAATCACGACCGTAGTGTCTACTTGCTCTTGAGTG
**adhE-R-down**	AAGACGCGCTGACAATAC
**pta-L-up**	ACTACGCGACGAAAGAAGAC
**pta-L-down**	GTTAACCGGCTTGCGCATAAACGCCTTTGCGTTCCATTG
**pta-R-up**	CAATGGAACGCAAAGGCGTTTATGCGCAAGCCGGTTAAC

**pta-R-down**	CAGAATGCGAAATGAGTGTG
**pl-L-up**	TCATTTAATACTGCCGCCTC
**pl-L-down**	GTGCTCAGTATCACCGCCAGTGGTATTTATGTCAACACCGCCGCTATGCAGAAAATTGCGCAC
**pl-R-up**	GGCGGTGTTGACATAAATACCACTGGCGGTGATACTGAGCACTGCAAGAGGGATAAAAAATG
**pl-R-down**	GTCAGGTTGCCAAGTGTGTC
**pl-yz-FP**	AATACCACTGGCGGTGATAC
**mgsA-L-up**	CAACACGCTGGCCGAAGT
**mgsA-L-down**	GCTCAGTATCACCGCCAGTGGTATTTATGTCAACACCGCCATCCAGTCGCCGCATTTC
**mgsA-R-up**	ACCCCTTGGGGCCTCTAAACGGGTCTTGAGGGGTTTTTTGCGTTCCACCCAGCTCATC
**mgsA-R-down**	CTCGCCATTACCTCAACTG
**hbd-up**	GGCGGTGTTGACATAAATACCACTGGCGGTGATACTGAGCACAGGGAGGTCTGTTTAATG
**hbd-down**	CATGACTAATCCTCCTAAAATTTATTTTGAATAATCGTAG
**crt-up**	CTACGATTATTCAAAATAAATTTTAGGAGGATTAGTCATG
**crt-down**	CAATCATATCATACCTCCAGCTATCTATTTTTGAAGCCTTC
**ter-up**	GAAGGCTTCAAAAATAGATAGCTGGAGGTATGATATGATTG
**ter-down**	CAAAAAACCCCTCAAGACCCGTTTAGAGGCCCCAAGGGGTTATGCTAGTTAAATCCTGTCGAACCTTTC
**hct-yz-mgsFP**	CCTTATTCACCAGGCGATTG
**hct-yz-mgsRP**	TGCCTGAGCAATTCCTGAAC
**pdmb-PpchA-pchA-up**	CGCGGTCAAAAAAATGCTAAGGCTGTTTTGGCGGATGAGA
**pdmb-PpchA-pchA-down**	GGTACGGATATATTCCTGTGCAGCTCATTTCAGAATATTTGCCAGAAC
**PpchA-pchA-pdmb-up**	GTTCTGGCAAATATTCTGAAATGAGCTGCACAGGAATATATCCGTACC
**PpchA-pchA-pdmb-down**	TCTCATCCGCCAAAACAGCCTTAGCATTTTTTTGACCGCG

**LEE1-32-GFP-up**	CATAACGGTTCTGGCAAATATTACTTGTACAGCTCGTCC
**LEE1-32-GFP-down**	TTGACATTTAATGATAATGTATTTTACACATTAAAGAAGGAGATATACATATGGTGAGCAAGGGCGAGG
**pdmb-AA-LEE1-32-up**	ACATTATCATTAAATGTCAATTCTGAAATGAGCTGCACAGGAATATATCCGTAC
**pdmb-AA-LEE1-32-down**	GGACGAGCTGTACAAGTAATATTTGCCAGAACCGTTATG
**pdmb-AA-GFP-LEE1-98-down**	GATAATGTATTTTACACATTATTTGTTTAACTTTAAGAAGG
**pdmb-AA-GFP-LEE1-98+152-up**	CTTCTTAAAGTTAAACAAAGCTTTAATATTTTAAGCT
**pdmb-AA-GFP-LEE1-98+152-down**	AGCTTAAAATATTAAAGCTTTGTTTAACTTTAAGAAG
**pdmb-AA-GFP-LEE1-98+218-up**	GAAGAAAATCATCTCGTTAATTCTGAAATGAGCTGCACAG
**pdmb-AA-GFP-LEE1-98+218-down**	CAAGTCCATACATTCAGCTTTTTGTTTAACTTTAAGAAG
**LEE1-98+218-up**	CTTCTTAAAGTTAAACAAAAAGCTGAATGTATGGACTTG
**LEE1-98+218-down**	CTGTGCAGCTCATTTCAGAATTAACGAGATGATTTTCTTC
**pdmb-AA-GFP-LEE1-379+152-up**	CATAAGCTACAGCCAGCACTTCTGAAATGAGCTGCACAG
**pdmb-AA-GFP-LEE1-379+152-down**	ATAGCTTAAAATATTAAAGCTTTGTTTAACTTTAAGAAGGAGATATACCATGGTGAGCAAGGGCGAGG
**LEE1-379+152-up**	CCTTCTTAAAGTTAAACAAAGCTTTAATATTTTAAGCTAT

**LEE1-379+152-down**	CTGTGCAGCTCATTTCAGAAGTGCTGGCTGTAGCTTATG
**LEE1-379+444-up**	CTTCTTAAAGTTAAACAAAGCTGTCGGCCTACGCCCGAC
**pdmb-AA-GFP-LEE1-379+444-down**	GTCGGGCGTAGGCCGACAGCTTTGTTTAACTTTAAGAAG
**pdmb-AA-GFP-LEE1-379+444-up**	CCTTCTTAAAGTTAAACAAATAATGTGTAAAATACATTATC

The red characters are pl promoter sequences. The underline parts indicated the N20 sequences.
